# Evidence for ontogenetically and morphologically distinct alternative reproductive tactics in the invasive Round Goby *Neogobius melanostomus*

**DOI:** 10.1371/journal.pone.0174828

**Published:** 2017-04-03

**Authors:** Katinka Bleeker, Karen de Jong, Nils van Kessel, Camilla A. Hinde, Leopold A. J. Nagelkerke

**Affiliations:** 1 Behavioural Ecology Group, Wageningen University and Research, Wageningen, The Netherlands; 2 Institute for Zoology, Research Station Grieterbusch, University of Cologne, D-50923 Cologne, Germany; 3 Bureau Waardenburg bv, AJ Culemborg, The Netherlands; 4 Radboud University Nijmegen, Institute for Water and Wetland Research. Department of Environmental Science, Nijmegen, The Netherlands; 5 Aquaculture and Fisheries Group, Wageningen University and Research, Wageningen, The Netherlands; University of Windsor, CANADA

## Abstract

Alternative reproductive tactics are characterized by the occurrence of discrete alternative morphs that differ in behavioural, morphological and physiological traits within the same sex. Although much effort has been made to describe the behaviour, morphology and physiology of such alternative morphs, less effort has been invested investigating how much overlap there is in the characteristics of such morphs in natural populations. We studied random population samples of the invasive Round Goby *Neogobius melanostomus* from five different localities in the river Rhine system in the Netherlands. We found two morphologically and physiologically distinct male morphs which likely represent alternative reproductive tactics. Almost all mature males under 9.35 cm total length had a gonadosomatic index > 3%, suggestive of a sneaker tactic, while nearly all males above 9.35 cm has a gonadosomatic index of < 3%, suggestive of a parental tactic. Cheek size and eye diameter alone were sufficient to distinguish the two morphs. Gonads had a different relationship with size in the two morphs, indicating separate growth trajectories. The gonad mass of sneaker morphs would be ca. 7.5 times as high as the gonad mass of parental morphs of the same total length after extrapolation. Few (9%) intermediates were found, suggesting that the expression of alternative reproductive tactics is determined before the first breeding season. This contrasts with studies on other goby species, which show evidence of plastic tactics that can be affected by social circumstances. We conclude that it is possible to distinguish two alternative male morphs in the Dutch Round Goby population using morphological measurements alone. Although behavioural observations are needed to provide conclusive evidence, the difference in GSI between these morphs indicates that these morphs reflect alternative reproductive tactics.

## Introduction

Alternative reproductive tactics where individuals use alternative ways to obtain fertilisations [1], are widespread among fish species [2,3]. Alternative reproductive tactics are characterized by a discontinuous distribution in behavioural, morphological and physiological traits between individuals of the same sex [4]. They are particularly common in male individuals, as a result of disruptive sexual selection [1,5]. In general, there are two types of male tactics: ‘parental’ (sometimes called conventional, bourgeois or type I) and ‘sneaker’ (parasitic or type II) tactics [4,6,7]. Parental males compete for, defend, and monopolize reproductive resources, while sneaker males exploit the investment of parental males [6,8,9]. Parental males are generally large, exhibit secondary sexual characteristics, court females, and take care of the eggs [7]. Sneaker males, on the other hand, are smaller (in length and weight), lack clear secondary sexual characteristics, and surreptitiously fertilize eggs in the nest of a parental male [7]. Sneaker males invest in reproduction over growth, as opposed to parental males [7], and gain a fitness pay-off by avoiding the costs of courtship and parental care [10,11]. However, sneaker males often have a lower fertilization success as compared to parental males and therefore possibly lower reproductive success [12,13].

Alternative reproductive tactics can be fixed, where conditions in early life determine which tactic is adopted [14], or plastic, in which individuals switch tactics during their lifetime [1]. Plastic tactics can be flexible and depend on the current condition of an individual in relation to the level of competition in the population [8,14]. Tactics can also follow an ontogenetic gradient, where younger males follow a sneaking tactic until they are large enough to become parental males [2,8,11,14–16]. Theory predicts that when alternative reproductive tactics evolve, both tactics should provide benefits in terms of reproductive output under certain conditions [1]. Where the fitness curves of the tactics cross, there should be a clear switch-point at which individuals change tactics [4]. Body size can be the main factor in this switch-point, and in many fish species sneaker males change to a parental tactic at a later life stage because the benefits of a sneaking tactic generally decrease with increasing body size, while the benefits of a parental tactic increase with body size [1,5]. In species of the Gobiidae, both forms of plastic tactics have been found [15–21]. Several goby species have been suggested to follow an ontogenetic gradient [15–17,19,21], however it was also found that, in the absence of competition, small goby males can switch to a parental tactic [18,20].

Alternative reproductive tactics and its general principles (i.e. density and condition dependency, proximate mechanisms) are widely studied (for example, see [7]). However, few studies have focussed on where tactic switch-points lie in natural populations. In the Dung Beetle, *Onthophagus taurus*, Hunt and Simmons [22] have shown that the point where males switch from hornless to horned coincided with the point where fitness gains (in terms of reproductive success) increased with body size. In fish, experimental studies have shown that social environment can affect whether a male reproduces as a sneaker or parental morph [18,20]. In Sand Goby, *Pomatoschistus minutus*, sneaker morphs were found to reproduce as a nest holder (parental morph) when competition with other males was low [20]. Furthermore, Black Goby, *Gobius niger*, sneaker morphs showed courtship behaviour, spawned and showed parental care for eggs when kept in a tank with only females present [18]. If switch-points are based on local conditions, we predict there to be large differences between localities in which individuals adopt certain tactics.

We measured morphological characteristics in a random population sample of Round Goby, *Neogobius melanostomus*, caught in five river locations within the River Rhine system in the Netherlands. The Round Goby is an important and potentially harmful invasive freshwater species, known for its wide-ranging invasion across North America, the Baltic Sea and European rivers [23]. In the Netherlands, this species was first recorded in 2004 [24] and has since displayed an invasive dispersal pattern [25,26]. There is strong evidence for the existence of male alternative reproductive tactics in Round Goby populations in the Great Laurentian Lakes [27]. However, evidence for the existence of male alternative reproductive tactics in Western European populations is still anecdotal. We expect distinct male morphs being present in European Round Goby populations as well. The aim of this study was to assess whether we could clearly distinguish different male morphs, indicating alternative reproductive tactics. Furthermore, we aim to assess whether there was overlap in morphological characteristics between these morphs, which would suggest flexibility in the tactic switch-point.

## Materials and methods

### Materials

**Fig 1:**
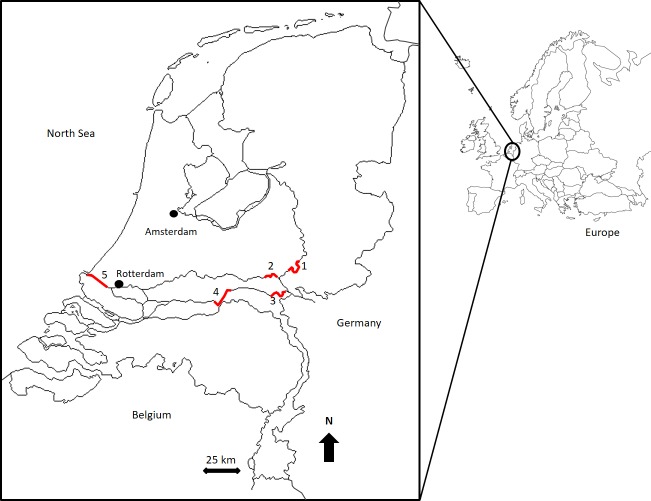
Position of five locations from the ‘Active Freshwater Fish Monitoring’ survey. These areas; Gelderse IJssel (*1*), Nederrijn (*2*), upstream Waal (*3*), downstream Waal (4) and Nieuwe Waterweg (*5*) are all within the river Rhine system.

The Round Goby population samples were provided from the ‘Active Freshwater Fish Monitoring’ survey in 2014, a legally required ecological status assessment relating to the Water Framework Directive (WFD; 2000/60/EC). This standardized survey records fish populations in permanent transects since 1997 and is carried out in 22 key areas. Frozen Round Goby samples were provided from this fish survey (sampled in March-April 2014) from five different localities (up to ± 175 km apart) within the river Rhine system (Fig 1). Round Goby samples were caught using electrofishing (generator pulsating D.C. 200–300 V) and bottom-trawling (smallest mesh size 20 mm). Both gear types were used in four river localities, electrofishing was not used in Nieuwe Waterweg as this gear type was not sufficient to use in this part of the river. See [28] for specific methods used in the ‘Active Freshwater Fish Monitoring’ survey.

### Fish samples

**Fig 2:**
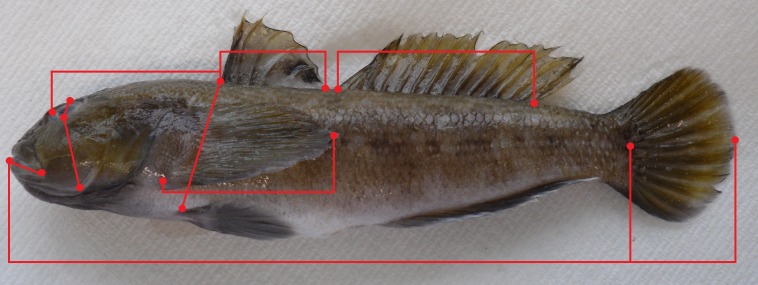
Schematic drawing of Round Goby, *Neogobius melanostomus*, with illustration of distance-based measurements (red dots) taken from the lateral view. The following morphometric characters were measured as follows: pre-orbital distance (PD, distance between the front of the upper lip to the orbit), eye diameter (ED, the greatest bony diameter of the orbit), lower jaw length (LJW, straight line measurement between the snout tip and posterior edge of mandible), cheek size (CS, males only: maximum depth measured from the lower edge of the orbit to the base of the gill slits), body depth (BD, maximum depth measured from the base of the 1^st^ dorsal fin to the pelvic fin), pectoral fin length (PFL, from the base to the tip of the pectoral fin), pre-dorsal fin length (PDL, front of the upper lip to the base of the 1^st^ dorsal fin), 1^st^ dorsal fin length (DFL1, from the base of the 1^st^ dorsal spine to the base of the last dorsal ray from the 1^st^ dorsal fin), and 2^nd^ dorsal fin length (DFL2, from the base of the 1^st^ dorsal spine to the base of the last dorsal ray from the 2^nd^ dorsal fin).

All individuals (*n* = 953) were measured (total length, TL, to the nearest mm) and weighed (wet total body weight, W_T_, to 0.1 g). Sex was determined for each individual through external examination of the urogenital papilla [23]. A random sample (*n* = 234) was subsequently taken from these individuals. Wet total eviscerated body weight (W_E_, to the nearest 0.1 g), and wet gonad mass (W_G_, to the nearest 0.1 g) were measured. In order to select mature individuals, a classification scale (Stage I to V, derived from [29]) with the macroscopic characteristics of the gonads was used to determine maturity. Individuals in stage IV and V were classified as mature. The colour (light, intermediate or dark brown) of each individual was recorded and mottledness (i.e. spots or patches of colour) was recorded as either present or absent. For further analysis of morphometric characters, a random sample of 128 male individuals was taken from the population sample. From these 128 males, 69 specimens could be classified as mature based on the classification scale only. Nine morphometric characters were measured (Fig 2) from mature individuals, to the nearest 0.1 mm from the left side of the body [30], using a digital calliper. Gonadosomatic index (GSI) was calculated as: GSI = (W_G_ / W_E_) x 100% [31], where W_G_ is wet gonad mass (testes and seminal vesicles) and W_E_ is wet total eviscerated body weight.

### Statistical analysis

Differences between sexes in total length, body weight and sex ratio and between male morphs in morphometric characters, or PCA-scores were tested with analysis of variance (ANOVA), or with Wilcoxon’s signed-ranked test, in case of violation of the assumptions of ANOVA [32]. *Post hoc* pairwise comparisons were performed with Tukey-Kramer’s least significant difference (LSD) tests [32]. A Loess regression [33] of GSI against total length was performed to estimate whether GSI changed at a certain total length. This local regression is a non-parametric method for estimating the regression surface [33]. The Loess regression indicated males could be divided in two groups based on length and GSI rather than indicating a continuous relation between length and GSI. Furthermore, a Piecewise regression was performed on the same data. Piecewise regression is used for nonlinear relationships where the data show different trends in different regions, to model the regression function in ‘pieces’, using linear regression [34]. To test at which total length these groups were separated, we used a simplified threshold regression which allows us to model the distinction between these groups at a certain threshold value [35]. The simplified threshold regression assumes that on either side of a particular value of total length, GSI values varied around a different mean. For a series of such cut-off points we calculated the mean value of the log-transformed GSI values and their sum of squares on both sides of the point. The cut-off point for which the total sum of squares of both sides was lowest was taken as the best estimator of the total length at which the groups were divided. Mature males larger than this total length were classified as ‘parental morph’, and males smaller were classified as ‘sneaker morph’. The association between the sneaker and parental morph and colour/mottledness was tested with Fisher’s exact tests. To investigate overall body shape in relation to these morphs, the nine external metrics were analysed by principal components analysis (PCA) [36]. To correct for size, Mosimann’s method was used [37,38], i.e. size was defined as the geometric mean of all metrics for every individual. All metrics were divided by this shape variable. The log of the resulting ratios was taken, to linearize the relationships between the original metrics. Before PCA, all variables were standardized to zero mean and standard deviation of 1. The relation of gonad mass and total length for the different male morphs were analysed with analysis of covariance (ANCOVA) [32]. All statistical analyses were performed in SAS (9.3 Software (SAS Institute INC., Cary, NC, USA).

## Results

Total length and body weight were analysed from a total of 387 males and 566 females of the Round Goby (Table 1). Males (40.6% of the total sample) were significantly larger (Wilcoxon signed-rank test, *n*_*1*_ = 566, *n*_*2*_ = 387, *W* = 97938, *P* < 0.05) and heavier (Wilcoxon signed rank test, *n*_*1*_ = 566, *n*_*2*_ = 387, *W* = 101190, *P* < 0.05) than females (59.4% of the total sample). Overall sex ratio was found to be female biased (1.46:1). Characteristics of the Dutch Round Goby population per river locality can be found in the supporting information (S1 Table).

**Table 1:** Characteristics in the Dutch Round Goby population from five localities within the river Rhine system.

	Sex	Mean	Standard error	Minimum	Maximum
Total length (cm)	M	8.52	0.13	3.80	14.90
	F	7.91	0.08	2.90	13.50
Body weight (g)	M	11.25	0.57	0.50	61.75
	F	8.10	0.25	0.19	41.33
Overall sex ratio	F: M	1.46: 1			

(F: female, *n* = 566; M: male, *n* = 387)

### Male alternative reproductive tactics

**Fig 3:**
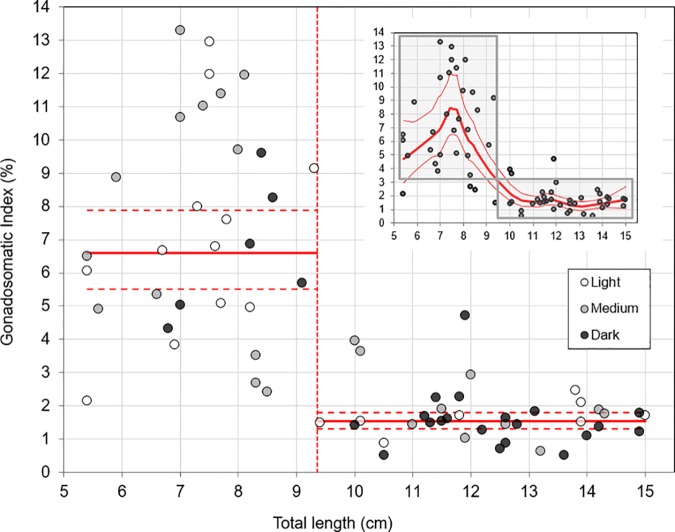
Piecewise and Loess (inset)-regressions of gonadosomatic index (GSI, %) vs. total length (TL, cm). Marker colours indicate light (open circle), medium (grey circle), and dark (black circle) Round Goby males.

**Fig 4:**
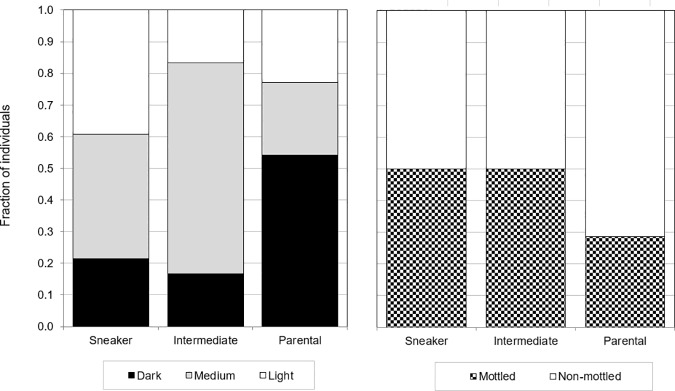
Association between reproductive groups and colour (dark, medium, light) and mottledness (present or absent) in Round Goby males. Reproductive groups are: sneaker morphs (*n* = 28), intermediates (*n* = 6) and parental morphs (*n* = 35).

A random sample of 128 male individuals was taken from the population sample. From these 128 males, 69 individuals were classified as mature, based on the classification scale only. A discontinuity between mature males was apparent from the Loess regression of GSI against total length (Fig 3). Threshold regression indicated a clear distinction at a total length of 9.35 cm. This point was used to classify sneaker morphs (GSI>3% and TL<9.35cm, *n* = 28) and parental morphs (GSI<3% and TL>9.35cm, *n* = 35). There were few intermediates present (*n* = 6). The pattern of high GSI values in sneaker morphs and low GSI values in parental morphs is consistent across all river locations (S1 Fig). A significant association was found between these three groups and body colouration (Fig 4), with parental morphs usually being darker (Fisher’s exact test, *P* = 0.031), while there was no such relationship with mottledness (*P* = 0.18).

**Fig 5:**
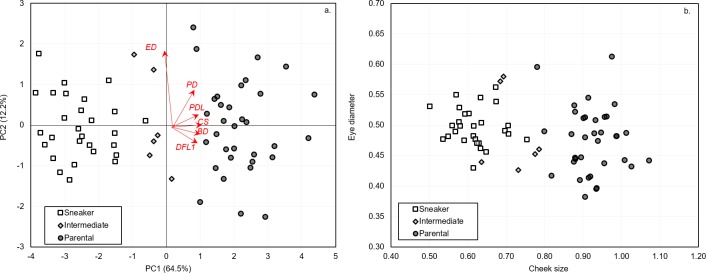
**Biplot of the first two principal component axes (a) and scatterplot of cheek size vs. eye diameter (b) in Round Goby males.** Based on 9 morphometric characters for sneaker morphs (*n* = 28), intermediates (*n* = 6) and parental morphs (*n* = 34; one parental was excluded from this analysis due to missing measurements).

The variation in external morphometric characters (Fig 2), was captured in a principal component analysis (PCA). The PCA explained more than 76% of this variation in the first two axes and uncovered a clear distinction between sneaker and parental morphs with intermediates being also intermediate for external morphometric characters (Fig 5A). PC1 was most strongly associated with cheek size (loading factor of 0.77), body depth (0.74), pre-dorsal fin length (0.72) and second dorsal fin length (0.72). PC2 was most strongly associated with eye diameter (1.76) and pre-orbital distance (0.83). There was a significant difference between the morphs on PC1 (ANOVA, *F*_(2,65)_ = 254.9, *P* < 0.001) and the two morphs differed significantly from each other and from intermediates (Tukey-Kramer’s LSD test, *P* < 0.001), with sneaker morphs showing the lowest and parental morphs the highest values. Higher values on PC1 represented a larger cheek size, body depth and pre-dorsal fin length. There was no significant difference between morphs on PC2 (ANOVA, *F*_(2,65)_ = 0.068, *P* = 0.935). Cheek size and eye diameter, significantly differed between morphs (ANOVA, cheek size: *F*_(2,65)_ = 99.93, *P* < 0.001; eye diameter: *F*_(2,65)_ = 26.4, *P* < 0.001) and their combination was found to be effectively discriminating between sneaker and parental morphs (Fig 5B).

### Gonad development

**Fig 6:**
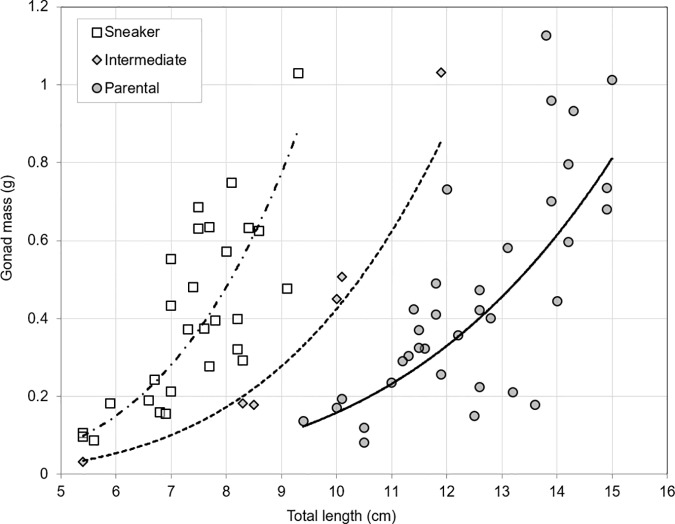
Gonad mass plotted against total length for Round Goby sneaker morphs (*n* = 28), intermediates (*n* = 6) and parental morphs (*n* = 35). Lines indicate fitted power curves.

Gonad mass increased significantly with total length (Fig 6: ANCOVA: *F*_(2,65)_ = 144.82, *P* < 0.0001), and there was also a significant effect of morph (*F*_(2,65)_ = 49.92, *P* < 0.0001). The relation of gonad mass with total length is of the form: *GW = a∙TL*^*b*^, in which *b* is the common slope (*b* = 4.03, with s.e. = 0.335) and *a* is a morph-specific constant (1.48∙10^−5^, 3.97∙10^−5^, and 11.1∙10^−5^ g∙cm^-1^ for parental morphs, intermediates, and sneaker morphs, respectively). Extrapolation indicates that the gonad mass of sneaker morphs would be ca. 7.5 times as high as the gonad mass of parental morphs of the same length.

## Discussion

In a random sample of Round Goby males from five locations in the Dutch river Rhine system, 91% of mature males belonged to two morphologically distinct male morphs that showed characteristics of parental (*n* = 35) and sneaker (*n* = 28) tactics. The parental morph was characterised by a TL>9.35 cm and a GSI<3%, while the sneaker morph had a TL<9.35cm and a GSI>3%. The pattern of low GSI values in parental morphs and high GSI values in sneaker morphs was consistent across all river locations. This suggests that there is a switch-point above which the parental tactic is more beneficial than the sneaker tactic. We found very few intermediates early in the breeding season, suggesting that tactics are mostly fixed before the start of the breeding season. Such a clear bimodal distribution is in line with theory on alternative tactics, but has not often been identified in random population samples, even if alternative tactics are very common in fish species [11].

In the Black Goby, Mazzoldi & Rasotto [39] have shown that, in the field, parental males were larger than 9 cm total length, while sneaker males ranged from 6–8 cm. In Grass Goby, *Zosterisessor ophiocephalus*, Scaggiante *et al*. [31] found an intermediate investment in both testes and seminal vesicles in males ranging between 12.6 to 16 cm total length. Possibly, these males are switching from one tactic to another, or adopt either a sneaking or parental tactic depending on opportunity. The above findings correspond to the cut-off point we found in this study. Body size, thus, seems to be one of the main factors in switching reproductive tactics [20] and it is likely that the benefits of adopting a parental tactic increase at a certain size range. In Round Goby, we found a bimodal distribution in a random population sample, and we were able to pinpoint the cut-off point at 9.35 cm total length, suggesting that size is a major factor for Round Goby males to switch tactics.

Parental morphs were larger in total length and had larger cheek sizes and smaller eye diameters compared to sneaker morphs, which would allow future studies to distinguish the morphs based on the measures from these characteristics. There was also a significant association between body colouration and reproductive tactics, although this relationship was not absolute with only 54% of parental morphs showing a dark colouration and 40% of the sneaker morphs being light. Colour, however, may be a biased measure as during catch fish appear dark regardless of tactic [40]. Our findings resemble results from a study on a Canadian invasive population of Round Goby, in which different reproductive male morphs are known to exist. These morphs primarily differed in colour and length [27], where large mature males with a dark to black colouration were considered as putative parental males, and small, juvenile- or female- shaped mature males, with a light, mottled pattern as putative sneakers [27]. Dark morphs were larger and heavier and had wider heads than light morphs, similar to our findings. Further, dark morphs invested twice as much in accessory glands as compared to light morphs, while the latter invested nearly three times as much in testes [27]. Our study suggests that in the Dutch Round Goby population the difference in gonad development is even larger, extrapolating from the correlations we found, gonads of putative sneakers would be ca. 7.5 times larger than those of parental males at the same total length. Also in other goby species, similar differences in colouration and body morphology were found [17,18,31,39]. For instance, in Black Goby, parental males were found to be larger and older than sneaker males and their body colouration differed, where parental males had a black nuptial colouration while sneaker males where paler [17]. In addition, the elongation of the fourth ray of the dorsal fin distinguished the morphs [39]. Immler *et al*. [18] found a higher mean GSI in Black Goby sneaker males as compared to parental males, which is also in line with our findings.

In species with indeterminate growth, like fishes, predictable changes in conditions (regarding physical or social environment of an individual, or its own physical condition [41]) occur with ontogeny or size, and a sequential expression (i.e. a fixed or reversible sequence) of reproductive tactics may be favoured [15,42–45]. Size variation occurs as fish continue to grow after reaching sexual maturity and smaller, younger males compete with larger, older males by adopting a sneaker tactic until they are large enough to become a parental male [2,3]. Male alternative reproductive tactics have been suggested to follow such an ontogenetic gradient in Common Goby, *Pomatoschistus microps* [15], Grass Goby [16], Black Goby [17], Two-spotted Goby, *Gobiusculus flavescens* [21], and possibly Sand Goby [19]. In case of an ontogenetic sequence (i.e. immature–sneaker–parental), intermediates are expected to be present in a relatively large proportion of the population. A high proportion of intermediates would suggest that switching between tactics is common during our sampling period at the start of the breeding season, while a low proportion would suggest more fixed tactics. Rasotto & Mazzoldi [17] found ca. 30% intermediates in the Black Goby, with intermediate size and intermediate elongation of the fourth first dorsal fin ray, and these were thought to behave as sneaker or parental males depending on opportunity. We, however, only found a small proportion of intermediates (9%). Fagundes et al. [46] also found a small proportion of intermediates or transient individuals (7%) in Peacock Blenny, *Salaria pavo*. In this long-term mark-recapture study, it was found that intermediates express a sneaker tactic and switch to the nest-holder or parental tactic within their first breeding season [46]. While we cannot tell when tactics are fixed, because we did not follow fish for any length of time, our data do suggest that tactics in the Round Goby are fixed before the breeding season. We found separate trajectories in gonad development between sneaker and parental Round Goby morphs which suggests that tactics may be determined in early life. However, fixed tactics are less common in fish than plastic tactics [2] and it is more likely that males can switch between breeding seasons. Our data are also consistent with a sequential tactic if sneaker morphs switch to a parental tactic between breeding seasons when they reach the cut-off size of 9.35 cm total length.

The expression of sneaker and parental tactics in the Peacock Blenny, were found to be affected by birth date, where males born early in the season became nest-holders (i.e. parentals) and were larger at the start of their first breeding season, as compared to males born later in the season [46]. This ‘birthdate effect’ is proposed in species where reproductive success is related to body size and where alternative reproductive tactics are dependent on environmental and/or physical conditions [11]. Individuals born early in the season have, as compared to those born later in the season, the advantage of a longer growth period and may also experience different environmental conditions during this growth period. Individuals born in the same year at different periods will thus have a different body size at the start of their first breeding season [11]. When born early in the season, individuals can become parental males at the start of their breeding season [46], or they can start by adopting a sneaking tactic [47,48]. In salmonids it was found that individuals that grow quicker and mature at an earlier age adopt a sneaking tactic (“jacks”) [48]. Fagundes *et al*. [46], on the other hand, found that the early born individuals in Peacock Blenny reach a larger size at the start of their first breeding season and consequently follow a nest-holder pathway, in which individuals reproduce as parentals in their first breeding season, while later born individuals follow a parasitic pathway, in which males reproduce as sneakers in their first breeding season and as nest-holder in subsequent season. However, males from both pathways showed similar growth curves, and males matured simultaneously but at a different body size in their first breeding season [46]. The Round Goby has an extended reproductive season, with multiple spawns during a season [49,50]. In its native range, spawning typically takes place every 3–4 weeks from April through September [51]. It is reasonable to assume that Round Goby offspring born throughout these months have a different body size at the start of their first breeding season depending on their birth date and differences in environmental conditions. The ‘birth date effect’, may thus be applicable to the invasive Dutch Round Goby population.

## Conclusion

This study is the first to describe population characteristics of the invasive Round Goby in the Netherlands, and provides evidence for the presence of male alternative reproductive tactics in this population. A clear cut-off point between sneaker and parental morphs at 9.35 cm total length was found with few intermediates present in the population. This suggests that the switch-point, where the fitness functions of the two tactics cross, is mainly dependent on fish size. We suggest that male alternative reproductive tactics in this population are largely fixed at the onset of the breeding season and may be affected by birth date. The separate relationships between total length and gonad mass in the two morphs may indicate separate life-history pathways in male sneaker and parental morphs.

## Supporting information

S1 Fig Bar graph of mean GSI (%, logarithmic scale) with confidence intervals given per river location and overall for both sneaker and parental morphs. Abbreviations indicate river locations as followed: All: all river locations pooled together, GIJ: Gelderse IJssel, N: Nederrijn, UW: upstream Waal, DW: downstream Waal, and NW: Nieuwe Waterweg.
(TIFF)

S1 File Round Goby population dataset and population subset of mature males. (XLSX)

S1 Table Characteristics of the Dutch Round Goby population per river site. (PDF)
