# Supplementary figures and images for: Evidence for ontogenetically and morphologically distinct alternative reproductive tactics in the invasive Round Goby Neogobius melanostomus
Source: PLoS One. 2017 Apr 3;12(4):e0174828. doi: 10.1371/journal.pone.0174828 (PMC5378390; doi:10.1371/journal.pone.0174828)

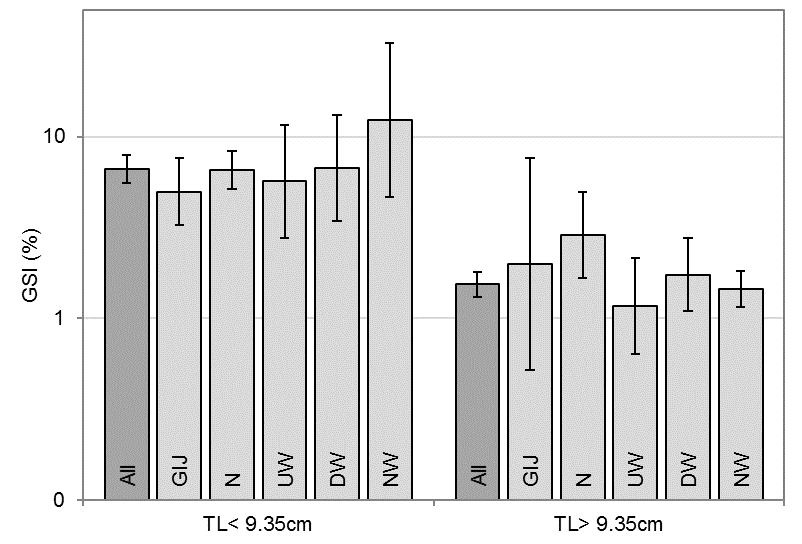

Supplement: S1 Fig — Abbreviations indicate river locations as followed: All: all river locations pooled together, GIJ: Gelderse IJssel, N: Nederrijn, UW: upstream Waal, DW: downstream Waal, and NW: Nieuwe Waterweg. (TIFF) [file pone.0174828.s001.tiff]
