# Supplementary material for: Evidence for ontogenetically and morphologically distinct alternative reproductive tactics in the invasive Round Goby Neogobius melanostomus
Source: PLoS One. 2017 Apr 3;12(4):e0174828. doi: 10.1371/journal.pone.0174828 (PMC5378390; doi:10.1371/journal.pone.0174828)
Supplement: S1 Table — (PDF) [file pone.0174828.s003.pdf]

S1 Table. Characteristics of the Dutch Round Goby population per river site.

	**Gelderse IJssel**		**Nederrijn**		**Upstream Waal**		**Downstream Waal**		**Nieuwe Waterweg**	
**Males**	74		204		84		188		16	
**Females**	31		134		72		119		31	
**F:M ratio**	1.58:1		2.39:1		1.52:1		1.17:1		0.52:1	
	**M**	**F**	**M**	**F**	**M**	**F**	**M**	**F**	**M**	**F**
**Total length (cm)**	8.47	8.51	8.16	7.75	9.29	8.63	7.85	7.47	10.94	8.51
**Body weight (g)**	10.2	10.32	8.51	6.89	15.30	11.49	9.00	6.90	23.4	9.71

(F: Female, M: male)
